# Uptake of the Siderophore Triacetylfusarinine C, but Not Fusarinine C, Is Crucial for Virulence of Aspergillus fumigatus

**DOI:** 10.1128/mbio.02192-22

**Published:** 2022-09-20

**Authors:** Mario Aguiar, Thomas Orasch, Yana Shadkchan, Patricia Caballero, Joachim Pfister, Luis Enrique Sastré-Velásquez, Fabio Gsaller, Clemens Decristoforo, Nir Osherov, Hubertus Haas

**Affiliations:** a Institute of Molecular Biology, Biocenter, Medical University of Innsbruck, Innsbruck, Austria; b Department of Clinical Microbiology and Immunology, Sackler School of Medicine Ramat-Aviv, Tel Aviv, Israel; c Department of Nuclear Medicine, Medical University Innsbruck, Innsbruck, Austria; Universidade de Sao Paulo

**Keywords:** fungi, molds, *Aspergillus fumigatus*, iron, siderophore, uptake, virulence

## Abstract

Siderophores play an important role in fungal virulence, serving as trackers for *in vivo* imaging and as biomarkers of fungal infections. However, siderophore uptake is only partially characterized. As the major cause of aspergillosis, Aspergillus fumigatus is one of the most common airborne fungal pathogens of humans. Here, we demonstrate that this mold species mediates the uptake of iron chelated by the secreted siderophores triacetylfusarinine C (TAFC) and fusarinine C by the major facilitator-type transporters MirB and MirD, respectively. In a murine aspergillosis model, MirB but not MirD was found to be crucial for virulence, indicating that TAFC-mediated uptake plays a dominant role during infection. In the absence of MirB, TAFC becomes inhibitory by decreasing iron availability because the mutant is not able to recognize iron that is chelated by TAFC. MirB-mediated transport was found to tolerate the conjugation of fluorescein isothiocyanate to triacetylfusarinine C, which might aid in the development of siderophore-based antifungals in a Trojan horse approach, particularly as the role of MirB in pathogenicity restrains its mutational inactivation. Taken together, this study identified the first eukaryotic siderophore transporter that is crucial for virulence and elucidated its translational potential as well as its evolutionary conservation.

## INTRODUCTION

With an estimated worldwide annual number of 16 million infections, mainly in immunocompromised patients, the saprobic fungus Aspergillus fumigatus is one of the most common airborne fungal pathogens in humans (1, 2). Notably, coronavirus disease 2019 (COVID-19)-associated pulmonary aspergillosis has emerged as a life-threatening complication in patients admitted to intensive care units (1, 3). The diagnosis of aspergillosis is problematic as it often lacks sensitivity and provides only late diagnosis. The gold standard still relies on the detection of serum galactomannan, a fungal cell wall component (4, 5), although many other diagnostic tools for aspergillosis are under development (6–9). Notably, siderophores (see below) were found to have great potential as biomarkers in blood, urine and bronchoalveolar lavage fluid for human A. fumigatus infections (6, 7, 10, 11) and for the *in vivo* imaging of A. fumigatus infections by ^68^Ga positron emission tomography (PET) (8, 12, 13). For the treatment of aspergillosis, only a few drug classes are currently used, which target either the fungal cell wall or the cell membrane, e.g., echinocandins, polyenes and azoles. However, clinical concerns arise because of their side effects and due to the emergence of resistant strains (1, 4, 14, 15). Consequently, there is a need for alternatives. One possibility for the development of novel antifungal drugs is a Trojan horse approach (16–18), in which toxic compounds are conjugated to siderophores for selective import into the pathogen, a concept that has been realized in a recently FDA-approved drug against bacterial infections (19). Noteworthy, in this respect, the A. fumigatus siderophore transporter Sit1 was found to mediate the uptake and, consequently, the antifungal activity of VL-2397, which resembles a ferrichrome-type siderophore (20).

The flexibility of Aspergillus in metabolism and nutrient acquisition confers a major advantage during infection, particularly under limited nutrient availability. As iron serves as an essential cofactor for numerous cellular processes, most successful pathogens rely on a meticulous equilibrium of the element iron as a virulence trait (21–23). A. fumigatus employs uncharacterized low-affinity ferrous iron uptake and two high-affinity iron acquisition systems, termed reductive iron assimilation (RIA) and siderophore-mediated iron acquisition (24). In RIA, ferric iron is first reduced to its ferrous form by a membrane-bound metal reductase, FreB, and then reoxidized and internalized by a protein complex consisting of the ferroxidase FetC and the iron permease FtrA (25, 26). Siderophores are low-molecular-mass ferric iron chelators differing in structure and categorized as hydroxamates, catecholates, carboxylates, phenolates and mixed types (27). *Ascomycetes* such as A. fumigatus exclusively produce hydroxamate class siderophores. Hydroxamates include rhodotorulic acid and ferrioxamine-, fusarinine-, coprogen- and ferrichrome-type siderophores. A. fumigatus secretes two fusarinine-type siderophores, triacetylfusarinine C (TAFC) and fusarinine C (FsC), to capture environmental iron. Moreover, it employs two ferrichrome-type siderophores, ferricrocin (FC) and hydroxyferricrocin, for the intracellular transport of iron in hyphae and conidia, respectively (28). The biosynthesis of hydroxamate class siderophores involves several enzymes and cellular compartments, with the first dedicated enzymatic step being the hydroxylation of ornithine (28, 29); the inactivation of the ornithine hydroxylase SidA blocks the biosynthesis of all siderophores and renders A. fumigatus avirulent in a murine model of aspergillosis (26). Subsequently, the pathways for the biosynthesis of extra- and intracellular siderophores split due to the transfer of different acyl groups to *N*^5^-hydroxyornithine, acetyl for intracellular siderophores and anhydromevalonyl for extracellular siderophores (30, 31). FsC and FC are then assembled by nonribosomal peptide synthetases, while TAFC is derived from the triple *N*^2^-acetylation of FsC catalyzed by SidG (30).

After the secretion and chelation of iron, the siderophore-iron complexes (ferriforms also labeled with [Fe]) are taken up by specific transporters belonging to the “siderophore-iron transporter” (SIT) subfamily of the major facilitator protein superfamily (32). SITs are found only in the fungal kingdom, and even species that lack siderophore biosynthesis, such as Saccharomyces cerevisiae, Candida albicans, Candida glabrata and Cryptococcus neoformans, possess SITs for the uptake of siderophores produced by other species, termed xenosiderophores (28). SITs are commonly composed of about 600 amino acids that fold into 14 transmembrane helices (33) and most likely function as proton symporters (21, 34, 35). The substrate specificity of SITs was first characterized in the siderophore nonproducer S. cerevisiae, which possesses four SITs (36). Phylogenetic analysis revealed that all S. cerevisiae SITs are more similar to each other than they are to SITs from other fungal species (32), which indicates that these transporters arose after the split of the *Saccharomycotina* clade from other members of the *Ascomycota*. Consequently, the substrate specificity of A. fumigatus SITs cannot be predicted on the basis of sequence similarity to S. cerevisiae SITs. A. fumigatus possesses five potential SITs (termed Sit1, Sit2, MirB, MirD and MirC), which are transcriptionally repressed by iron, which is indicative of a role in iron homeostasis (37). Recently, Sit1 and Sit2 were found to be essential for the utilization of coprogen type and ferrichrome-type siderophores, displaying both redundancy and exclusivity depending on the ferrichrome type (38, 39). Moreover, Sit1 was shown to be the exclusive transporter for ferrioxamine-type xenosiderophores (39), which are produced by bacteria (40, 41). The heterologous expression of the Aspergillus nidulans and A. fumigatus siderophore transporter-encoding genes in S. cerevisiae indicated that MirB transports TAFC (42, 43). However, this approach does not allow conclusions regarding the exclusivity of substrate specificity or metabolic function in A. fumigatus. MirC was found to be localized intracellularly, most likely in vacuole-like structures, and has been suggested to participate in FC biosynthesis (44), while MirD’s function has remained elusive to date. Notably, not all members of the SIT family transport siderophores; i.e., S. cerevisiae Gex2 and Schizosaccharomyces pombe Str3 have been reported to transport glutathione and heme, respectively (45, 46).

Due to the roles of siderophores in virulence and in the imaging of fungal infection and their potential therapeutic applications via the coupling of toxic compounds in a Trojan horse approach (24), the characterization of the exact substrate specificities of the A. fumigatus SITs is not only of scientific but also of translational interest. Therefore, the goal of this study was to elucidate the substrate specificities of A. fumigatus MirB and MirD and to analyze their roles in physiology, virulence and the uptake of a fluorescent TAFC conjugate.

## RESULTS

### Generation of gene deletion mutants for functional characterization of MirB and MirD.

To functionally characterize MirB and MirD, the encoding genes (*mirB* [AFUA_3G03640/AFUB_044500] and *mirD* [AFUA_3G03440/AFUB_044810]) were deleted in A. fumigatus A1160^+^ (termed the wild type [WT]) by replacement with the hygromycin (*hph*) and pyrithiamine (*ptrA*) resistance marker genes, respectively, resulting in the Δ*mirB* and Δ*mirD* strains. To avoid interference with endogenous siderophore production, *mirB* and *mirD* were also deleted by applying the same approach in a selection-marker-free A. fumigatus mutant strain lacking siderophore biosynthesis (Δ*sidA*), resulting in the Δ*sidA* Δ*mirB* and Δ*sidA* Δ*mirD* strains. A. fumigatus Δ*sidA* background strains are unable to grow in iron-depleted medium without supplementation with utilizable siderophores (47), which allows the characterization of siderophore uptake by simple growth studies. As a further control, the *mirD* deletion was combined with the inactivation of both siderophore biosynthesis and RIA by generating the Δ*sidA* Δ*ftrA* Δ*mirD* mutant strain, which additionally avoids potential interference with RIA. To ensure the monitoring of gene deletion-specific effects, the deleted genes were reintegrated into the deletion mutants, resulting in the *mirB^c^*, *mirD^c^*, Δ*sidA mirB^c^*, Δ*sidA mirD^c^* and Δ*sidA* Δ*ftrA mirD^c^* strains. Phenotyping of the fungal strains was conducted using solid minimal medium with and without supplementation with iron, siderophores, or bathophenanthroline sulfonate (BPS), a ferrous iron-specific chelator that blocks RIA, thereby rendering siderophore-mediated iron uptake the only high-affinity mechanism (26).

### Utilization of TAFC by A. fumigatus is mediated exclusively by MirB.

**FIG 1:**
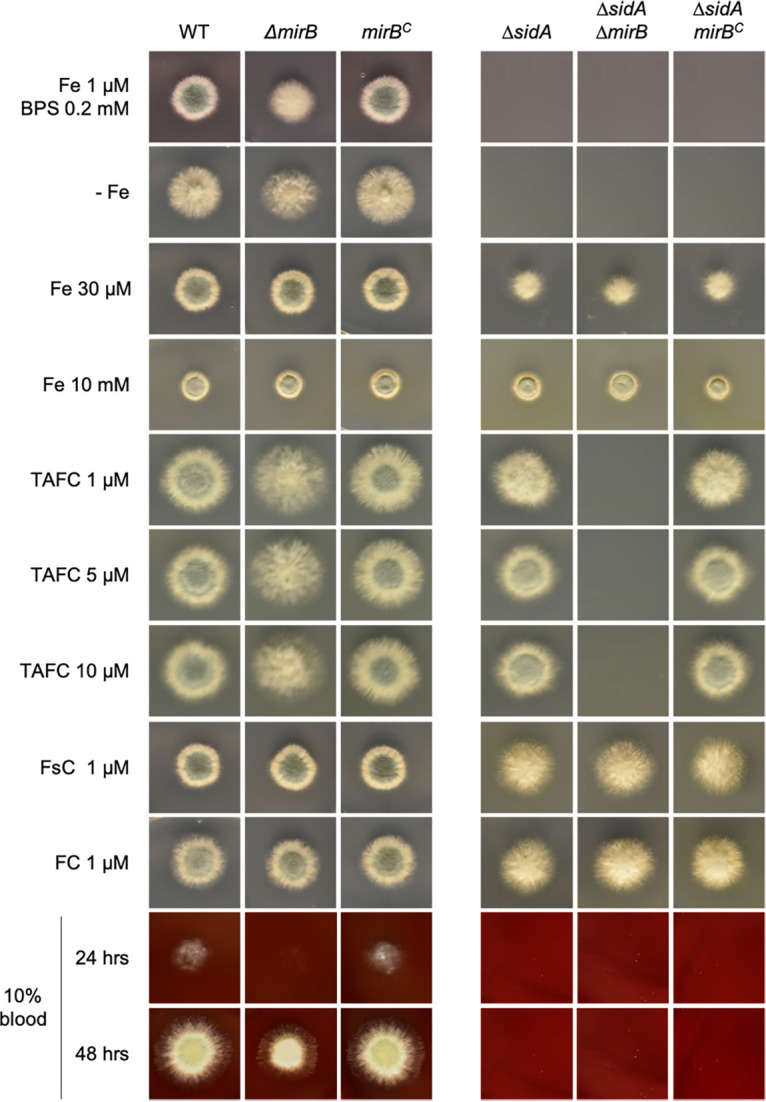
Utilization of TAFC by A. fumigatus is mediated exclusively by MirB. Four hundred conidia of fungal strains were point inoculated onto solid minimal medium supplemented with different concentrations of Fe^2+^, ferric siderophores, and BPS to analyze the role of MirB in the utilization of siderophores. The plates were incubated at 37°C for 48 h.

As shown in Fig. 1, the deletion of *mirB* (Δ*mirB*) resulted in reduced growth and sporulation on solid medium supplemented with 1 μM iron and 0.2 mM BPS as well as on iron-depleted (−Fe) (without iron addition) medium but not on medium containing higher concentrations of iron, such as 30 μM and 10 mM. These data indicated a role for MirB in adaptation to iron starvation. Supplementation with the siderophores ferricrocin and FsC but not TAFC cured the −Fe growth defect, which suggests a role for MirB in the uptake of TAFC but not the uptake of FsC and FC. As previously shown (26), the inactivation of siderophore biosynthesis (Δ*sidA*) blocked the growth of A. fumigatus in the presence of BPS as well as under −Fe conditions, and the deletion of *mirB* in this background (Δ*sidA* Δ*mirB*) prevented growth in the presence of 1, 5, and 10 μM TAFC but not with FsC or FC supplementation (Fig. 1). The reintegration of the *mirB* gene (*mirB^c^*) into Δ*mirB* strains cured the growth defects in all genetic backgrounds. Taken together, these data demonstrate that MirB indeed functions as a siderophore transporter in A. fumigatus and exclusively mediates TAFC utilization, while it does not play a major role in the uptake of FsC and FC. In agreement with the latter finding, FC was recently shown to be internalized by Sit1 and Sit2 (39). Consequently, the residual growth of the Δ*mirB* strain under −Fe conditions is most likely supported by FsC utilization and RIA. Notably, the lack of MirB (Δ*mirB*) decreased the growth of A. fumigatus on blood agar (Fig. 1), indicating an important role of TAFC in the utilization of iron sources present in the blood. As shown previously (26), the inactivation of siderophore biosynthesis (Δ*sidA*) blocked the growth of A. fumigatus on blood agar (Fig. 1), which reflects the importance of siderophores for the utilization of blood iron sources in general.

### Utilization of FsC by A. fumigatus is mediated mainly by MirD.

**FIG 2:**
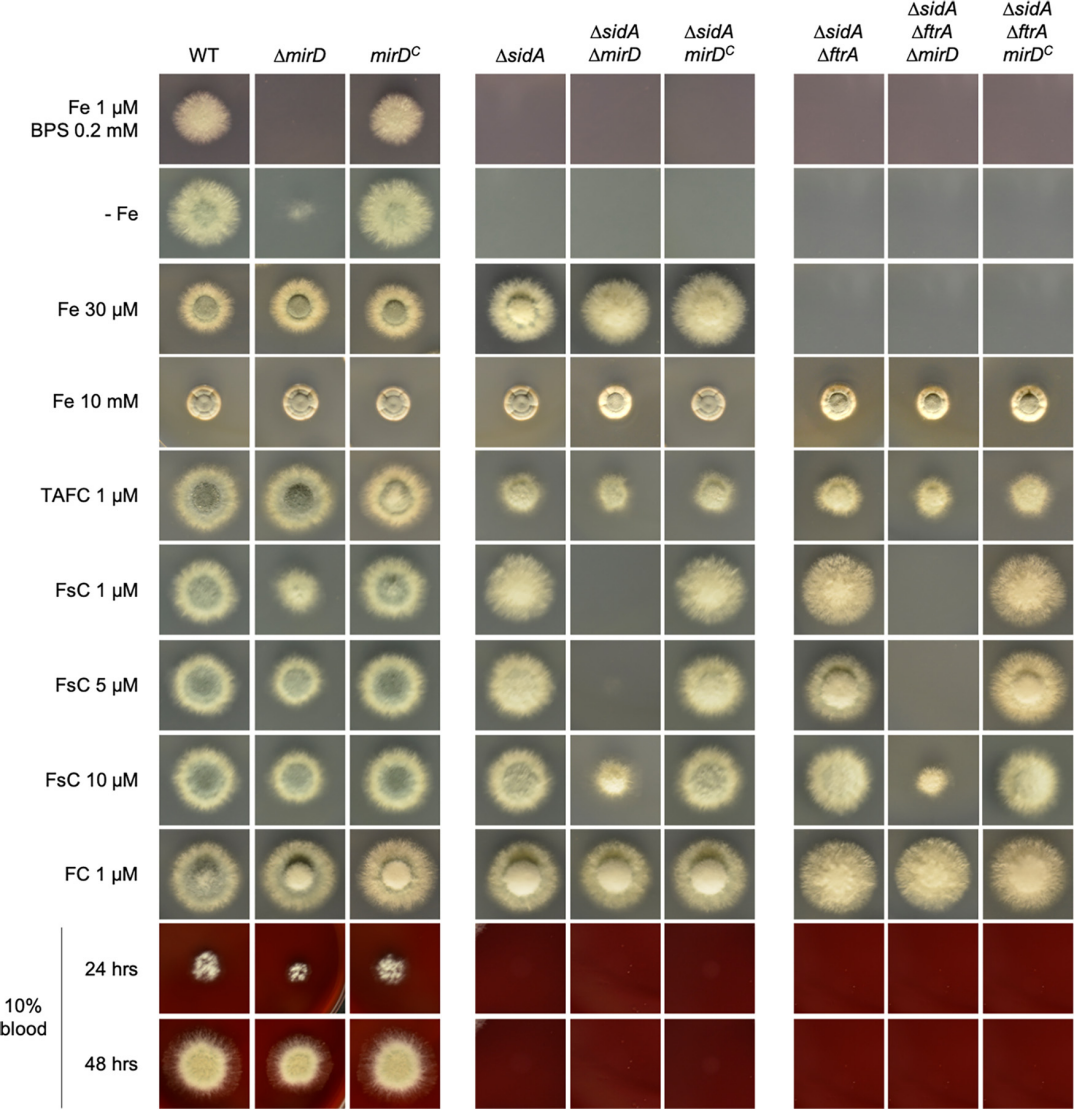
Utilization of FsC by A. fumigatus is mediated mainly by MirD. Four hundred conidia of fungal strains were point inoculated onto solid minimal medium supplemented with different concentrations of Fe^2+^, ferric siderophores, and BPS to analyze the role of MirB in the utilization of siderophores. The plates were incubated at 37°C for 48 h.

As shown in Fig. 2, the deletion of *mirD* (Δ*mirD*) resulted in a lack of growth on solid minimal medium containing 1 μM iron plus 0.2 mM BPS and reduced growth without iron supplementation (−Fe), which indicated a role for MirD in adaptation to iron starvation. With 30 μM and 10 mM iron supplementation as well as with TAFC or FC supplementation, the Δ*mirD* strain phenocopied the WT. Particularly with 1 μM but also with 5 and 10 μM FsC supplementation, the Δ*mirD* strain displayed reduced growth, indicating a role for MirD in FsC utilization. In the absence of endogenous siderophore production, the loss of MirD (Δ*sidA* Δ*mirD*) disabled growth on medium supplemented with 1 and 5 μM FsC and significantly decreased growth in the presence of 10 μM FsC but did not affect growth in medium supplemented with TAFC, FC, or high concentrations of iron (Fig. 2). As previously reported (39, 43), the simultaneous inactivation of siderophore biosynthesis and RIA (Δ*sidA* Δ*ftrA*) blocked growth in the presence of iron at a concentration of up to 2 mM. Also, in this genetic background, the loss of MirD (Δ*sidA* Δ*ftrA* Δ*mirD*) disabled growth on medium supplemented with 1 and 5 μM FsC but allowed limited growth with 10 μM FsC supplementation. Taken together, these data demonstrate that MirD functions as the main transporter of FsC. However, limited uptake is mediated by other transporters, as evidenced by the limited growth of the Δ*mirD* strain on 10 μM FsC even in the absence of siderophore production (Δ*sidA*) or siderophore production and RIA (Δ*sidA* Δ*ftrA*). On blood agar, the Δ*mirD* strain displayed slightly decreased radial growth but not as pronounced as that of the Δ*mirB* strain (Fig. 2).

### A. fumigatus SITs are conserved in other fungal species.

Phylogenetic analysis (48) of 38 SITs from 12 fungal species demonstrated that the five iron-regulated members of the A. fumigatus SIT family belong to different subclades (20). As previously discussed (32), all SIT family members of the *Saccharomycotina* species S. cerevisiae, C. albicans and C. glabrata are closely grouped, which indicates a common origin after the split from the other species despite having partially different substrates. Remarkably, despite overlapping substrate specificities, Sit1 and Sit2 are only distantly related, while MirB and MirD are localized in sister clades, indicating coevolution. The latter finding might be related to the fact that TAFC is derived from FsC, requiring only a single enzymatic step, i.e., triacetylation catalyzed by SidG (30). Among the species analyzed, MirB was found to be conserved in Aspergillus lentulus, A. nidulans and Fusarium oxysporum.

### Northern blot analysis confirms iron repression of *mirB* and *mirD*.

**FIG 3:**
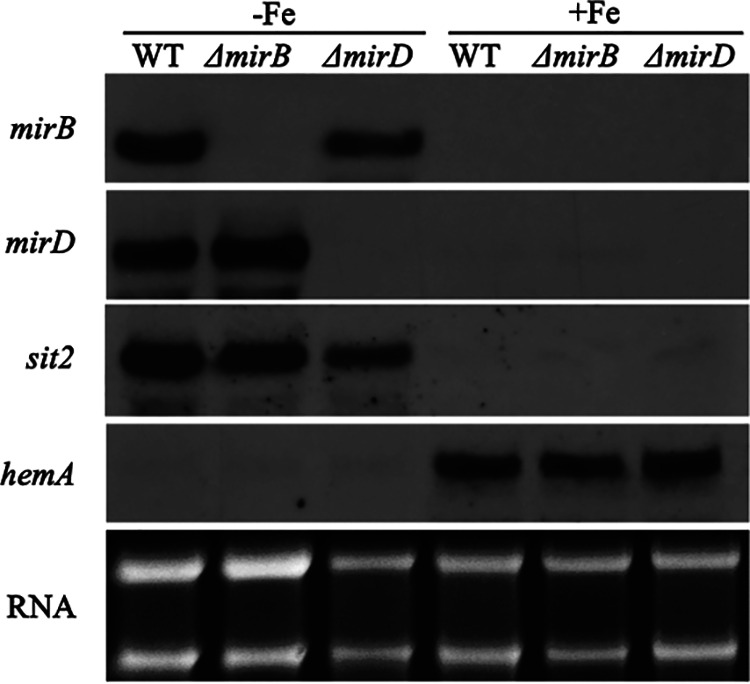
The genes encoding MirB and MirD are transcriptionally repressed by iron and their expression is missing in the respective gene deletion mutants. Fungal strains were grown for 17 h under iron starvation (−Fe) and iron sufficiency (+Fe); subsequently, total RNA was isolated and analyzed by Northern blotting for the expression of the indicated genes. Ethidium bromide-stained RNA served as a control for the loading and quality of RNA.

The genes encoding MirB and MirD were previously found to be localized in two gene clusters (AFUA_3G03390/AFUB_044860 to AFUA_3G03440*/*AFUB_044810 and AFUA_3G03640/AFUB_044500 to AFUA_303670*/*AFUB_044470) that are transcriptionally repressed by iron and contain other siderophore metabolic genes (37). The Northern blot analysis shown in Fig. 3 confirmed the iron repression of *mirB* and *mirD* at the transcript level and the absence of the respective transcripts in the Δ*mirB* and Δ*mirD* mutant strains, confirming their successful deletion. The iron repression of a previously analyzed iron-repressed gene encoding the siderophore transporter Sit2 (AFUA_7G04730/AFUB_091650), which is not localized in these two gene clusters (39), and the iron-induced *hemA* gene (AFUA_5G06270/AFUB_053800), encoding aminolevulinic acid synthase (49), confirmed the cellular iron state of the analyzed mycelia.

### Short-term uptake assays of ^68^Ga-labeled siderophores confirm that MirB and MirD mediate the uptake of TAFC and FsC, respectively.

To further confirm the substrate specificity of MirB and MirD, we conducted short-term uptake assays of ^68^Ga-labeled siderophores. We used mycelia grown under iron starvation, as the expression of the encoding genes is repressed by iron (see above) (37). In agreement with the results of the growth assays (Fig. 1), the loss of MirB impaired the uptake of [^68^Ga]TAFC but not [^68^Ga]FsC, and inversely, the loss of MirD impaired the uptake of FsC but not TAFC (Table 1). As the growth assays indicated the exclusive uptake of [^68^Ga]TAFC by MirB, the minimal siderophore uptake found in the Δ*mirB* mutant strain might result from the cross-chelation of ^68^Ga by endogenously produced FsC or might represent nonspecific TAFC binding to the cell wall. The same might apply to the minimal FsC uptake found in the Δ*mirD* mutant; however, here, the growth studies indicated low, MirD-independent FsC uptake. Taken together, these results confirm the substrate specificities of MirB and MirD identified in the growth assays.

**TABLE 1:** MirB- and MirD-mediated uptake of TAFC and FsC, respectively^*a*^

Strain	Mean uptake ± SD			
	[^68^Ga]TAFC		[^68^Ga]FsC	
	cpm	% of total radioactivity	cpm	% of total radioactivity
WT	68,338 ± 4,553	19.2 ± 1.3	59,047 ± 5,438	15.4 ± 1.4
Δ*mirB*	5,982 ± 2,162	1.7 ± 0.6*	61,136 ± 1,807	15.9 ± 0.5
Δ*mirD*	69,886 ± 11,219	19.6 ± 3.1	5,749 ± 1,105	1.5 ± 0.3*

a Values display the mean values for the short-term uptake of ^68^Ga-labeled siderophores ± standard deviations from four biological replicates, showing the measured counts per minute (cpm) and the percentages of the total radioactivity. *, *P < *0.01. Individual measurements can be found in Table S4 in the supplemental material.

10.1128/mbio.02192-22.5 TABLE S4 Short-term uptake of TAFC and FsC in the WT, Δ*mirD* and Δ*mirD* strains. The analysis included four biological replicates. cpm, gamma counts per minute. Download Table S4, DOCX file, 0.01 MB. Copyright © 2022 Aguiar et al. 2022 Aguiar et al. https://creativecommons.org/licenses/by/4.0/ This content is distributed under the terms of the Creative Commons Attribution 4.0 International license.

### The loss of MirB blocks the uptake of [Fe]DAFC-FITC in A. fumigatus.

**FIG 4:**
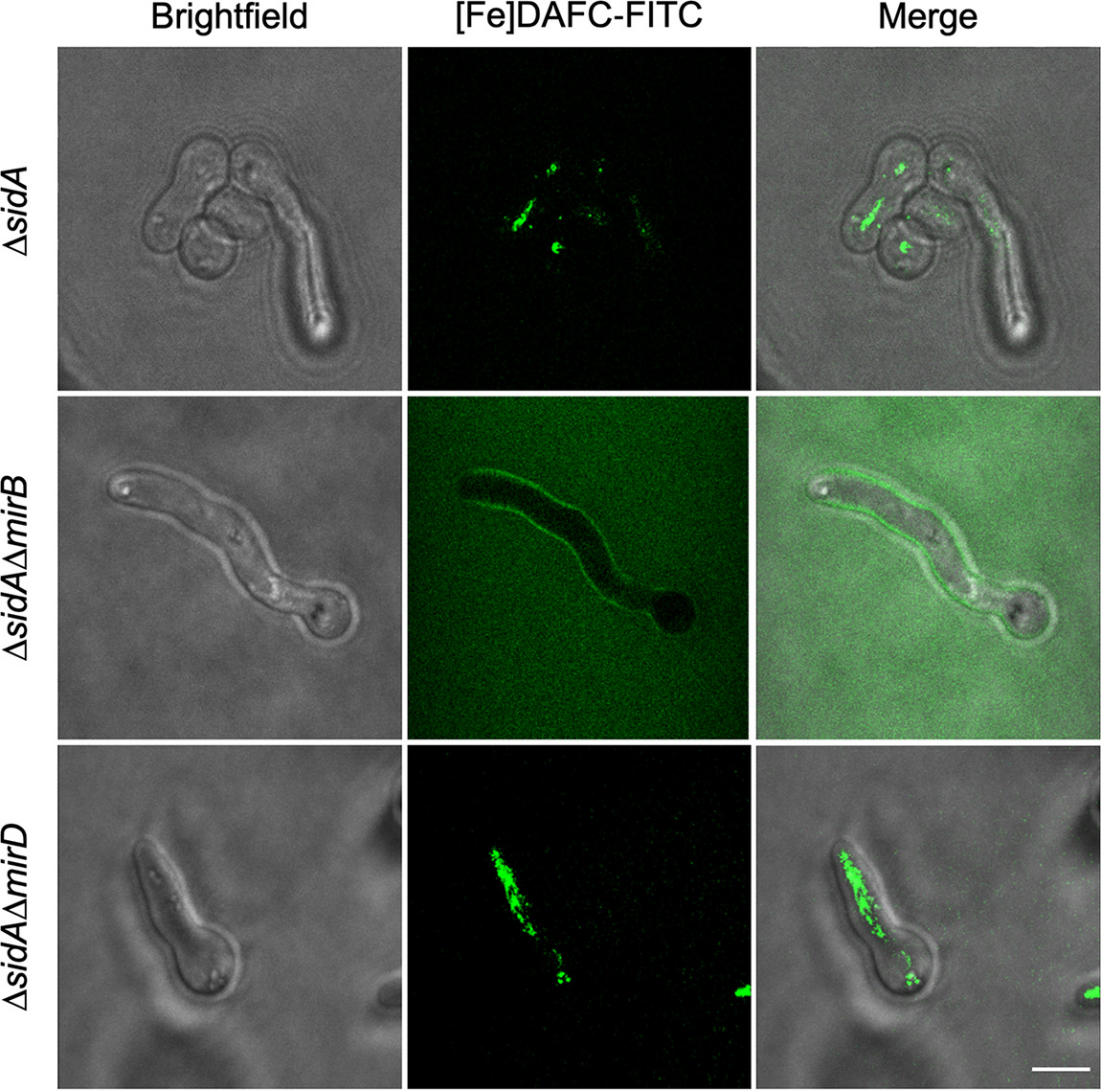
Loss of MirB disrupts the uptake of [Fe]DAFC-FITC in A. fumigatus. Fungal strains were grown in iron-depleted minimal medium for 16 h and subsequently incubated with 5 μM [Fe]DAFC-FITC for 30 min before imaging by epifluorescence microscopy. Bar, 10 μm. Pictures were taken with the same settings (not auto), indicating that the increased ambient fluorescence of the Δ*sidA* Δ*mirB* strain compared to the Δ*sidA* and Δ*sidA* Δ*mirB* strains reflects the lack of [Fe]DAFC-FITC uptake.

Previous studies indicated that A. fumigatus internalizes several fluorescent and nonfluorescent TAFC derivatives, including fluorescent [Fe]DAFC-FITC (16–18). Epifluorescence microscopy demonstrated that the internalization of [Fe]DAFC-FITC depends on MirB but not MirD (Fig. 4). These studies were conducted in a siderophore biosynthesis-lacking genetic background (Δ*sidA*) to increase iron starvation and, consequently, siderophore uptake. Notably, the genetic inactivation of TAFC uptake (Δ*mirB*) resulted in not only higher ambient fluorescence but also a slight accumulation of [Fe]DAFC-FITC on the hyphal surface, i.e., the cell wall and/or cell membrane. Taken together, these data demonstrate that MirB-mediated transport tolerates the derivatization of its substrate and that [Fe]DAFC-FITC serves as a TAFC surrogate.

### The loss of MirB or MirD has no major impact on siderophore production, but TAFC becomes inhibitory in the absence of MirB.

**FIG 5:**
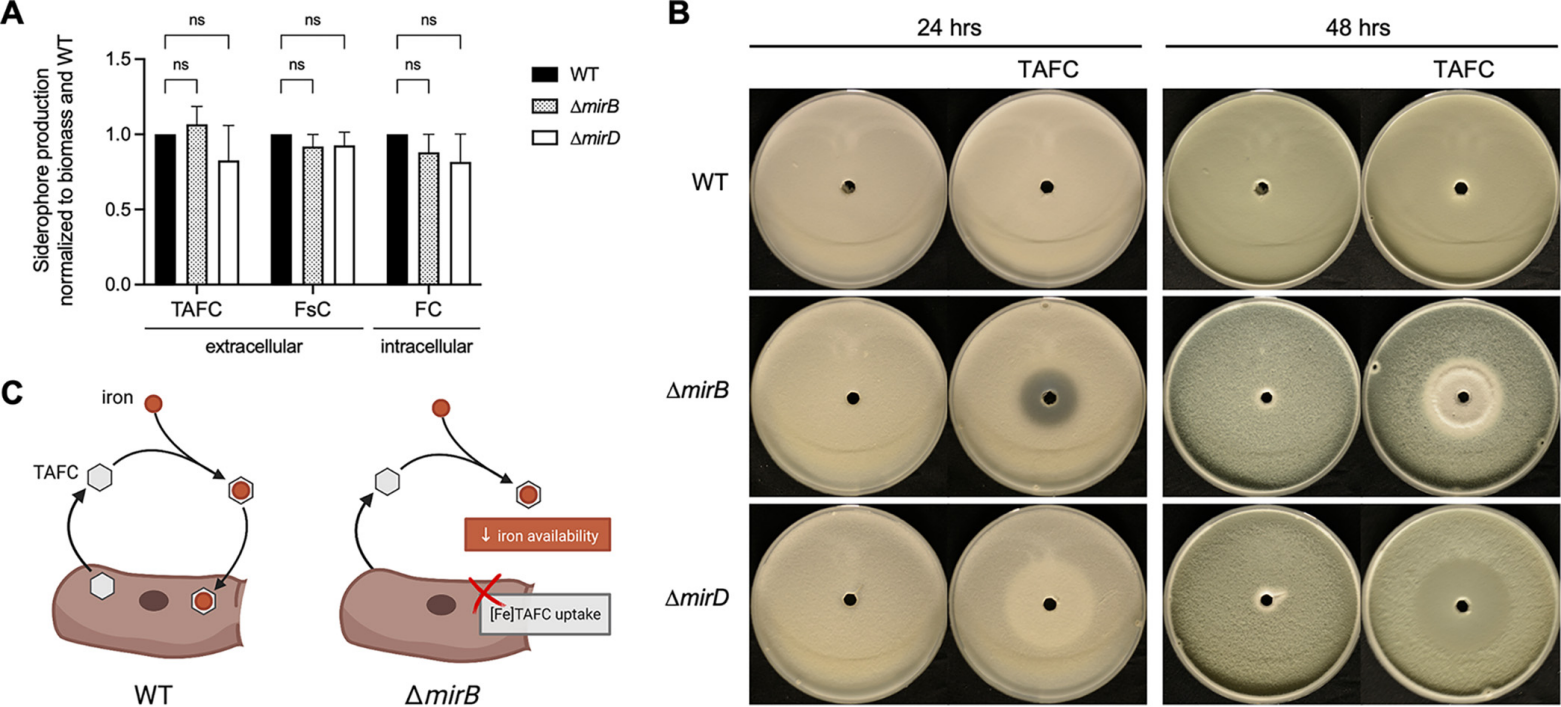
Inactivation of MirB causes growth inhibition of A. fumigatus and inhibition by TAFC. (A) The production of extracellular (TAFC and FsC) and intracellular (FC) siderophores remains unaffected upon the loss of MirB or MirD. Siderophore production was analyzed after growth for 24 h in liquid cultures in biological triplicates, normalized first to the respective biomass and then to the WT. The differences were not statistically significant (ns) (*P < *0.01). (B) To analyze the potential inhibition of A. fumigatus by TAFC, plates containing 25 mL of Aspergillus minimal medium were inoculated homogeneously with 2 × 10^7^ spores of the WT, Δ*mirB* and Δ*mirD* strains. Fifty microliters of 2 mM TAFC was added to a pricked hole in the center of each plate and the plate was incubated at 37°C for 48 h. Inhibition halo zones were seen in Δ*mirB* but not WT or Δ*mirD* plates, confirming the inhibition. This effect is fungistatic but not fungicidal, as indicated by the limited growth without sporulation in the 24-h inhibition zone after 48 h. The central halo zones visible in Δ*mirD* plates are partly due to growth promotion by the added TAFC. (C) Graphical representation of the potential autoinhibitory effect of TAFC in the absence of MirB. TAFC secreted by A. fumigatus chelates ambient iron and is utilized after iron chelation by WT strains (left), while the lack of MirB makes A. fumigatus “blind” to TAFC-chelated iron, which decreases iron availability (right).

The loss of either MirB or MirD did not affect the endogenous production of extracellular (TAFC and FsC) and intracellular (FC) siderophores by A. fumigatus (Fig. 5A). The growth assays shown in Fig. 1 indicated that MirB is the sole transporter of TAFC, and consequently, A. fumigatus is predicted to be “blind” to TAFC-chelated iron; i.e., it is unable to recognize this siderophore. As a further consequence, TAFC (the iron-free form) is predicted to inhibit the Δ*mirB* strain, as it is expected to decrease iron availability. Agar diffusion assays proved that TAFC is indeed inhibitory to A. fumigatus Δ*mirB* but not the WT or the Δ*mirD* mutant (Fig. 5B). This effect is fungistatic rather than fungicidal, as indicated by the limited growth without sporulation in the 24-h zone of inhibition after 48 h. The production and secretion of TAFC by the Δ*mirB* strain (Fig. 5A) suggest that TAFC production might have an autoinhibitory effect in the absence of MirB by decreasing iron availability (Fig. 5C). These data also underline that MirB is indeed the exclusive transporter of TAFC. FsC was not found to exhibit an inhibitory effect on the Δ*mirD* strain (data not shown), which agrees with MirD-independent FsC utilization, as also indicated by the growth assays (Fig. 2).

### MirB but not MirD is important for virulence in a murine aspergillosis model.

**FIG 6:**
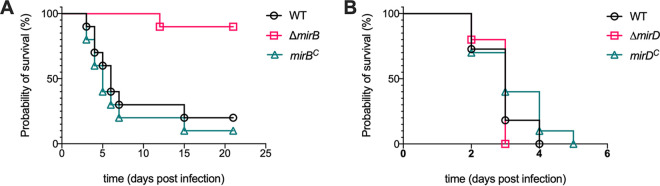
MirB but not MirD is important for the virulence of A. fumigatus. Survival plots of cortisone acetate-immunocompromised mice infected intranasally with A. fumigatus WT, Δ*mirB* and *mirB^c^* strains (A) and WT, Δ*mirD* and *mirD^c^* strains (B) are shown.

To analyze whether MirB and/or MirD plays a role in the virulence of A. fumigatus, we used a pulmonary model of infection in mice. Therefore, groups of 10 immunocompromised mice were infected with either the WT, Δ*mirB*, Δ*mirD*, *mirB^c^*, or *mirD^c^* strain (Fig. 6). The WT, the *mirB^c^* and *mirD^c^* reconstituted mutant strains, as well as the Δ*mirD* mutant strains displayed similar high mortality rates of 70 to 100% within 7 days postinfection. In contrast, the Δ*mirB* strain showed significantly reduced virulence (*P < *0.001) in infected mice, with a survival rate of 90%, even at 21 days postinfection. These results clearly indicate the importance of MirB for the virulence of A. fumigatus. In sum, these results demonstrate that TAFC-mediated uptake plays a dominant role during infection.

## DISCUSSION

Iron acquisition is an essential prerequisite for eukaryotic life. In most fungal species, including A. fumigatus, the main mechanism of iron uptake is via endogenous and exogenous siderophores (28). A. fumigatus can utilize a variety of hydroxamate class siderophores, including several ferrichrome types, endogenously secreted fusarinine-type FsC and TAFC, several ferrioxamines, as well as coprogen types, although the latter is utilized only poorly (39). In contrast, A. fumigatus was found to be unable to utilize the hydroxamate class siderophores basidiochrome and rhodotorulic acid, the catecholate class siderophore enterobactin, the carboxylate class siderophore rhizoferrin, and the mixed-class siderophores ornibactin and schizokinen (39). The A. fumigatus genome encodes seven potential SITs. Transcriptional repression by iron indicated a function in iron homeostasis for five of them, termed Sit1, Sit2, MirB, MirD and MirC (37). Sit1 and Sit2 were found to be essential for the utilization of coprogen types and ferrichrome types, displaying both redundancy and exclusivity depending on the ferrichrome type (39); e.g., the uptake of the ferrichrome-type antifungal VL-2397 depends exclusively on Sit1 (20). Of all analyzed ferrichrome types, only endogenously produced ferricrocin was shown to be poorly utilized independently of Sit1 and Sit2. Furthermore, Sit1 was shown to be the exclusive transporter of ferrioxamines, which are produced exclusively by bacteria (39). Here, we demonstrated by growth assays of the respective mutant strains in siderophore-producing and -lacking genetic backgrounds as well as by short-term uptake assays using ^68^Ga as an Fe surrogate that the acquisition of TAFC depends exclusively on MirB and that the uptake of FsC depends mainly on MirD. This study has substantially complemented the characterization of siderophore uptake by A. fumigatus, as for every siderophore shown to be utilized by A. fumigatus, a corresponding major transporter has been identified. Consequently, these results imply that the other A. fumigatus members of the SIT family do not play a major role in siderophore uptake. In agreement, MirC was found to be localized intracellularly, most likely in vacuole-like structures and has been suggested to participate in FC biosynthesis (44). Moreover, the gene encoding another potential SIT, CrmC (AFUA_3G13670/AFUB_035520), is localized in an isocyanide biosynthetic gene cluster and was previously shown to be regulated by copper but not iron (50). The function of the remaining potential SIT, AFUA_3G01360/AFUB_047020, remains elusive and previous studies indicated that its expression is not regulated by iron (37, 51). Notably, two other members of the SIT family from other species were shown to have substrates other than siderophores, as S. cerevisiae Gex2 and S. pombe Str3 have been reported to transport glutathione and heme, respectively (45, 46).

Previously, Sit1 and Sit2 were found to be dispensable for murine virulence (38), which appears plausible as these SITs mediate the uptake of xenosiderophores, which play no role in systemic infection. As shown here, MirB but not MirD is crucial for virulence in a murine model of pulmonary aspergillosis, indicating that TAFC-mediated iron uptake plays a dominant role during infection. The loss of MirB not only results in the defective uptake of TAFC but additionally might cause an autoinhibitory effect, as TAFC was shown to be inhibitory in the absence of MirB by decreasing the bioavailability of environmental iron due to chelation by a now-futile siderophore. In other words, this defect might support the host in its efforts to fight the pathogen via the deprivation and sequestration of iron, a condition termed anemia of inflammation (52, 53). Therefore, MirB appears to be a particularly attractive target for the development of novel antifungal strategies. In this respect, it is interesting that TAFC was found to exhibit antibiotic activity against a range of bacterial species, which are obviously not able to utilize this siderophore (54). Consequently, TAFC production most likely plays an additional role in microbial competition.

In the present study, epifluorescence microscopy revealed that MirB-mediated transport tolerates the conjugation of fluorescent FITC to TAFC (DAFC-FITC). Moreover, previous studies indicated that A. fumigatus internalizes several other fluorescent and nonfluorescent TAFC derivatives (16–18). These results emphasize the translational potential of SITs for the development of a novel antifungal drug mechanism based on the conjugation of toxic compounds to siderophores. Based on our results, TAFC conjugates appear most promising, as the importance of MirB in pathogenicity restrains its mutational inactivation and, consequently, the development of resistance at the level of uptake. Moreover, our data demonstrate that DAFC-FITC serves as a TAFC surrogate, allowing the analysis of TAFC uptake by epifluorescence microscopy. The elucidation of the substrate specificity of fungal siderophore transport is also helpful for the further development of siderophore-mediated *in vivo* imaging of fungal infections by ^68^Ga positron emission tomography (PET), as shown previously with TAFC, desferrioxamine B, desferrioxamine E and fluorescent derivatives thereof (8, 12, 13). The elucidation of the substrate specificities of SITs will assist in the structural characterization of substrate discrimination. The latter is supported by phylogenetic analyses of fungal SITs, which revealed that homologs of Sit1, Sit2, MirB, MirD, and MirC are members of subclades of the SIT family; the newly identified substrate specificities now improve the tentative functional prediction of homologs.

Collectively, this study functionally characterized the two missing A. fumigatus transporters for siderophore uptake, identified the first eukaryotic SIT that is crucial for virulence and indicated the autoinhibition of A. fumigatus during TAFC production via iron deprivation caused by MirB inactivation.

## MATERIALS AND METHODS

### Growth conditions.

For spore production and plate growth assays, A. fumigatus strains were grown on Aspergillus minimal medium containing 1% (wt/vol) glucose and 20 mM glutamine as the carbon and nitrogen sources, respectively (55). For iron-replete conditions, FeSO_4_ was added to the desired final concentration, while for iron starvation, iron was omitted. For the spore production of A. fumigatus lacking siderophore biosynthesis (Δ*sidA*) and/or RIA (Δ*ftrA*), an iron concentration of 3.0 mM was used (39). The ferrous iron chelator BPS (bathophenanthroline disulfonic acid disodium salt) (Sigma-Aldrich, Milwaukee, WI, USA) was used to block RIA (26). Supplementation with siderophores was done during the pouring of medium at 60°C. For agar plate point inoculation, 400 spores were used per inoculum. Liquid medium was inoculated with 10^6^ spores/mL using 100 mL of Aspergillus minimal medium in 0.5-L Erlenmeyer flasks with shaking at 200 rpm. Solid and liquid cultures were incubated at 37°C.

### A. fumigatus strains and their generation.

All studies were performed using A. fumigatus strain A1160^+^ (56), termed the WT here, which is a derivative of the clinical strain A. fumigatus CEA10 lacking nonhomologous recombination (Δ*akuB*^KU80^) to facilitate genetic manipulation (57, 58). All further strains used in this study are shown in Table S1 in the supplemental material; the plasmids and primers used for the generation of these strains are listed in Table S2. The transformation of A. fumigatus was performed as described previously (59, 60).

10.1128/mbio.02192-22.2 TABLE S1 A. fumigatus strains used in this study. Download Table S1, DOCX file, 0.02 MB. Copyright © 2022 Aguiar et al. 2022 Aguiar et al. https://creativecommons.org/licenses/by/4.0/ This content is distributed under the terms of the Creative Commons Attribution 4.0 International license.

10.1128/mbio.02192-22.3 TABLE S2 Plasmids and primers used for the generation of A. fumigatus mutant strains. Download Table S2, DOCX file, 0.02 MB. Copyright © 2022 Aguiar et al. 2022 Aguiar et al. https://creativecommons.org/licenses/by/4.0/ This content is distributed under the terms of the Creative Commons Attribution 4.0 International license.

A selection-marker-free Δ*sidA* mutant strain lacking endogenous siderophore biosynthesis was generated in A1160^+^ as previously described for A. fumigatus AfS77 (39). Briefly, the plasmid pΔ*sidA*-rec (39), which contains 1.0 kb of the 5′ and 3′ noncoding regions (NCRs) of *sidA* (AFUA_2G07680/AFUB_023720) flanking a self-excising hygromycin deletion cassette, was used for *sidA* deletion. Selection for Δ*sidA* transformants was performed using Aspergillus minimal medium containing 0.1 mg/mL hygromycin B (Calbiochem, San Diego, CA, USA). Mutant strains were confirmed by Southern blotting, as shown in Fig. S1A. Subsequently, the resistance cassette was excised from Δ*sidA* mutants by cultivation with 1% xylose (39).

10.1128/mbio.02192-22.1 FIG S1 Genomic organization and Southern blot analysis of the gene deletion mutants. (A) *sidA* locus in the WT and Δ*sidA* strains. The digestion of genomic DNA with NcoI resulted in a 2,735-bp fragment for the WT and a 6,844-bp fragment for Δ*sidA* mutants. Southern blot analysis using 3′-DIG probes confirms the deletion of Δ*sidA* strains. The digoxigenin-labeled hybridization probe was PCR amplified using the oligonucleotides listed in Table S3 in the supplemental material. (B) *ftrA* locus in the WT and Δ*ftrA* strains. The digestion of genomic DNA with KpnI and NotI resulted in a 4,491-bp fragment for the WT and an 8,024-bp fragment for Δ*ftrA* mutants. Southern blot analysis using 3′-DIG probes confirms the deletion of Δ*ftrA* strains. The digoxigenin-labeled hybridization probe was PCR amplified using the oligonucleotides listed in Table S3. (C) *mirB* locus in the WT and Δ*mirB* strains. The digestion of genomic DNA with EcoRI and BamHI resulted in a 4,874-bp fragment for the WT and a 4,147-bp fragment for the Δ*mirB* and Δ*sidA* Δ*mirB* strains. Southern blot analysis using 5′-DIG probes confirms the deletion of Δ*mirB* strains. The digoxigenin-labeled hybridization probe was PCR amplified using the oligonucleotides listed in Table S3. (D) *fcyB* locus in the WT and reconstituted *mirB^c^* strains. The digestion of genomic DNA with NcoI resulted in an 8,040-bp fragment for the WT and a 5,414-bp fragment for complemented *mirB^c^* strains. The digoxigenin-labeled hybridization probe was PCR amplified using the oligonucleotides listed in Table S3. (E) *mirD* locus in the WT, Δ*mirD*, Δ*sidA* Δ*mirD* and Δ*sidA* Δ*ftrA* Δ*mirD* strains. The digestion of genomic DNA with PstI resulted in a 3,596-bp fragment for the WT and a 6,454-bp fragment for the Δ*mirD*, Δ*sidA* Δ*mirD* and Δ*sidA* Δ*ftrA* Δ*mirD* mutant strains. The digoxigenin-labeled hybridization probe was PCR amplified using the oligonucleotides listed in Table S3. (F) *fcyB* locus in the WT and reconstituted *mirD^c^* strains. The digestion of genomic DNA with XbaI resulted in a 5,231-bp fragment for the WT and an 11,060-bp fragment for the complemented *mirD^c^* strains. The digoxigenin-labeled hybridization probe was PCR amplified using the oligonucleotides listed in Table S3. Download FIG S1, PDF file, 1.0 MB. Copyright © 2022 Aguiar et al. 2022 Aguiar et al. https://creativecommons.org/licenses/by/4.0/ This content is distributed under the terms of the Creative Commons Attribution 4.0 International license.

RIA was inactivated in the WT, Δ*sidA* and Δ*mirD* strains by replacing *ftrA* (AFUA_5G03800/AFUB_052310) with the hygromycin resistance cassette (*hph*). Therefore, the Δ*ftrA* deletion locus of a previously described mutant was PCR amplified by employing the primer pair MA105/MA106 and used for transformation (26) to yield the Δ*ftrA*, Δ*sidA* Δ*ftrA* and Δ*sidA* Δ*ftrA* Δ*mirD* strains (Fig. S1B and E).

For the deletion of *mirB* (AFUA_3G03640/AFUB_044500) in the WT and Δ*sidA* strains, plasmid pΔAFUmirB was generated, which contained the *mirB* 5′ NCR, a hygromycin resistance cassette (*hph*), and the *mirB* 3′ NCR, individually PCR amplified with oligonucleotide pairs oAfpUC19L_mirB5′.f/oAfmirB5′_hph.r, oAfmirB5′_hph.f/oAfmirB3′_hph.r and oAfmirB3′_hph.f/oAfpUC19L_mirB3′.r, respectively, using genomic DNA as the template for the NCRs and the plasmid pAN7.1 for the hygromycin cassette (61). The resulting fragments were assembled by a NEBuilder reaction (NEBuilder HiFi DNA assembly; New England BioLabs, Ipswich, MA, USA) with a pUC19L backbone (Thermo Fisher, Waltham, MA, USA). The *mirB* gene was deleted in the WT and Δ*sidA* strains by a bipartite marker technique (62), in which incomplete but overlapping fragments of the hygromycin resistance cassette flanked by the 5′ and 3′ NCRs of *mirB* were PCR amplified from plasmid pΔAFUmirB by employing primer pairs oAfpUC19L_mirB5′.f/ohph14 and ohph15/oAfpUC19L_mirB3′.r. Transformants were selected on minimal medium containing 0.1 mg/mL hygromycin B, resulting in the Δ*mirB* and Δ*sidA* Δ*mirB* mutant strains. Mutant strains were confirmed by Southern blotting, as shown in Fig. S1C.

For the complementation of *mirB* in the Δ*mirB* and Δ*sidA* Δ*mirB* strains, *mirB*, including flanking regions mediating its expression, was reintegrated into the *fcyB* locus (AFUA_2G09860/AFUB_025700) by utilizing the 5-flucytosine counterselectable marker approach (63, 64), resulting in the *mirB^c^* and Δ*sidA mirB^c^* strains. Therefore, the 5′ and 3′ NCRs of *fcyB* were PCR amplified with primer pairs *fcyB*1/*fcyB*-2RV and *fcyB*3/*fcyB*-4RV, and the *mirB* coding region, including 1.5 kb of the 5′ and 3′ NCRs, was amplified with primer pair AfMirBc-FW/AfMirBc-RV. The resulting PCR fragments were fused by a Gibson assembly reaction (Gibson assembly master mix; New England BioLabs, Ipswich, MA, USA). Subsequently, the reintegration construct was PCR amplified with the nested primer pair *fcyb*N1/*fcyb*N2 and used for transformation. Transformants were selected on minimal medium plates at pH 5.0 with 10 μg/mL 5-flucytosine (TCI, Eschborn, Germany). The correct genomic integration was verified by Southern blotting, as shown in Fig. S1D.

For the deletion of *mirD* (AFUA_3G03440/AFUB_044810) in the WT, Δ*sidA* and Δ*sidA* Δ*ftrA* strains, plasmid pMA10 was generated, which contained the *mirD* 5′ NCR, *ptrA* and the *mirD* 3′ NCR, individually PCR amplified with oligonucleotide pairs MA39/MA40, MA41/MA42 and MA43/MA44, respectively, by using genomic DNA as the template for the NCRs. Plasmid pSK275 (65) was used for the amplification of the *ptrA* cassette. The resulting fragments were assembled by a NEBuilder reaction (NEBuilder HiFi DNA assembly; New England BioLabs, Ipswich, MA, USA) with a pUC19L backbone (Thermo Fisher). For transformation, the deletion construct was PCR amplified from plasmid pMA10 with primers MA39 and MA44. Transformants were selected on minimal medium containing 0.1 mg/mL pyrithiamine (Calbiochem, San Diego, CA, USA), resulting in the Δ*mirD*, Δ*sidA* Δ*mirD* and Δ*sidA* Δ*ftrA* Δ*mirD* mutant strains, which were then confirmed by Southern blot analysis, as shown in Fig. S1E.

For the complementation of *mirD* in the Δ*mirD* strain, *mirD* was reintegrated into the *fcyB* locus of the Δ*mirD*, Δ*sidA* Δ*mirD* and Δ*sidA* Δ*ftrA* Δ*mirD* strains, resulting in the *mirD^c^*, Δ*sidA mirD^c^* and Δ*sidA* Δ*ftrA mirD^c^* strains. Therefore, the *mirD* gene, including the 1.5-kb 5′ and 3′ NCRs, was PCR amplified from genomic DNA using primer pair MA177/MA178 and integrated into the pUC19L-*fcyB* vector (63) containing the 5′ and 3′ NCRs of *fcyB*. The resulting plasmid, pMA20, was linearized by NotI digestion and used for transformation. The selection of transformants was carried out on minimal medium plates at pH 5.0 with 10 μg/mL 5-flucytosine. The correct genetic manipulations were proven by Southern blotting, as shown in Fig. S1F.

### Northern blot analysis.

Total RNA was isolated according to the Tri reagent (Sigma-Aldrich, Milwaukee, WI, USA) method by using peqGOLD phase trap reaction tubes (Peqlab, Erlangen, Germany). Formaldehyde agarose gels were used to separate 10 μg of total RNA, which was then blotted onto Amersham Hybond N^+^ membranes. Hybridization with the appropriate digoxigenin (DIG)-labeled probes was performed as previously described (66). The primers used for PCR amplification of hybridization probes can be found in Table S3.

10.1128/mbio.02192-22.4 TABLE S3 Probe primers used for Southern blot and Northern blot analyses. Download Table S3, DOCX file, 0.01 MB. Copyright © 2022 Aguiar et al. 2022 Aguiar et al. https://creativecommons.org/licenses/by/4.0/ This content is distributed under the terms of the Creative Commons Attribution 4.0 International license.

### Siderophores and radiolabeling of iron-free siderophores.

Triacetylfusarinine C and fusarinine C were produced and isolated in-house from iron-starved liquid A. fumigatus cultures as described previously (30, 66). The iron-free siderophores TAFC, FsC and FC were labeled for uptake assays as previously described (13, 67–69), by using 200 μL of a ^68^GaCl_3_ eluate (~20 to 30 MBq) obtained by the fractionated elution of a ^68^Ge/^68^Ga generator (IGG100; Eckert and Ziegler Isotope Products, Berlin, Germany) with 0.1 M hydrochloric acid. Ten microliters of iron-free siderophores (5 to 8 nmol) was incubated with the ^68^GaCl_3_ eluate in 40 μL of sodium acetate (NaOAc) buffer (pH 4.0) for 15 min at room temperature.

### Uptake assays with ^68^Ga-labeled siderophores.

The short-term uptake of ^68^Ga-labeled siderophores was performed as described previously (18, 67, 68). In short, A. fumigatus strains were cultivated in liquid iron-depleted Aspergillus minimal medium for 17 h (concentrated by gravimetric settlement) and 180 μL of these cultures was used in a phosphate-buffered saline (PBS)-prewashed 96-well MultiScreen HTS filter plate (1-μm glass fiber filter; Merck Millipore, Darmstadt, Germany). Subsequently, 50 μL of ^68^Ga-radiolabeled siderophores (~80 nM) was added along with 25 μL of PBS, and the mixture was incubated for 45 min at 37°C. Following incubation, the hyphae were washed twice with ice-cold Tris (15 mM) buffer and dry filter radioactivity was measured with a gamma counter.

### Epifluorescence microscopy for analysis of [Fe]DAFC-FITC uptake.

The fluorescent TAFC derivative DAFC-FITC was prepared as described previously by Pfister et al. (68). For epifluorescence microscopy, 1 × 10^5^ conidia were point inoculated onto iron-depleted minimal medium supplemented with 5 μM [Fe]DAFC-FITC and incubated at 37°C for 16 h to generate fungal germlings. Samples for microscopy were prepared by applying the “inverted agar method” (70, 71). Confocal laser scanning microscopy was performed using an HC Plan Apo 40×/1.10 CS2 water immersion objective on an SP8 confocal microscope (Leica Microsystems, Wetzlar, Germany) equipped with an 80-MHz pulsed white light laser (WLL). Images of DAFC-FITC (excitation, 495-nm WLL; emission, 515 to 530 nm) were processed using Leica Application Suite X (LAS X) and ImageJ/FIJI software.

### Murine model of invasive pulmonary aspergillosis.

Six-week-old female ICR mice were immunocompromised by two subcutaneous injections with 300 mg/kg of body weight of cortisone acetate, given 3 days before infection and on the day of infection. The mice were anesthetized by intraperitoneal (i.p.) injection with 100 mg/kg ketamine–10 mg/kg xylazine and infected intranasally with 5 × 10^5^ dormant spores/mouse, suspended in 20 μL of 0.2% Tween 20 in a saline solution (0.9% [wt/vol] NaCl), with 10 μL in each nostril. Endpoints for sacrifice included a drop of >15% in body weight or signs of acute distress. Mice were monitored for up to 21 days. Animal studies were done in accordance with Tel Aviv University institutional policies. The protocol was approved by the Minister of Health (MOH) Animal Welfare Committee, Israel (protocol number MOH 01-17-035). All efforts were made to minimize the number of animals used and animal suffering.

### Statistical analysis.

Data and statistical analyses were performed with the GraphPad Prism 9 software package (GraphPad Software Inc., San Diego, CA, USA). Analysis of variance (ANOVA) was used for significance testing of two groups. Differences between the groups were considered significant at a *P* value of *≤*0.05. Mortality results were analyzed by the log rank test for Kaplan-Meier survival curves, but a *t* test was used for significance testing of two groups. Differences between groups were considered significant at a *P* value of *≤*0.05.
