# Supplementary material for: Uptake of the Siderophore Triacetylfusarinine C, but Not Fusarinine C, Is Crucial for Virulence of Aspergillus fumigatus
Source: mBio. 2022 Sep 20;13(5):e02192-22. doi: 10.1128/mbio.02192-22 (PMC9600649; doi:10.1128/mbio.02192-22)
Supplement: TABLE S4 [file mbio.02192-22-s0005.docx]

|  | **TAFC** | | **FsC** | |
| --- | --- | --- | --- | --- |
|  | [cpm] | Uptake [%] | [cpm] | Uptake [%] |
| **WT** | 72967 | 20.5% | 66561 | 17.3% |
|  | 69847 | 19.6% | 59332 | 15.4% |
|  | 68404 | 19.2% | 56084 | 14.6% |
|  | 62134 | 17.4% | 54210 | 14.1% |
| Mean | 68338 | 19.2% | 59047 | 15.4% |
| STD | 4553 | 1.3% | 5438 | 1.4% |
| STD [%] | 0.07 | 6.7% | 0.09 | 9.2% |
| **∆*mirB*** | 6617 | 1.9% | 62730 | 16.3% |
|  | 4414 | 1.2% | 58646 | 15.3% |
|  | 4134 | 1.2% | 62152 | 16.2% |
|  | 8764 | 2.5% | 61014 | 15.9% |
| Mean | 5982 | 1.7% | 61136 | 15.9% |
| STD | 2162 | 0.6% | 1807 | 0.5% |
| STD [%] | 0.36 | 36.1% | 0.03 | 3.0% |
| **∆*mirD*** | 77409 | 21.7% | 7213 | 1.9% |
|  | 57248 | 16.1% | 5251 | 1.4% |
|  | 81053 | 22.8% | 5902 | 1.5% |
|  | 63835 | 17.9% | 4631 | 1.2% |
| Mean | 69886 | 19.6% | 5749 | 1.5% |
| STD | 11219 | 3.1% | 1105 | 0.3% |
| STD [%] | 0.16 | 16.1% | 0.19 | 19.2% |
