# Supplementary material for: Uptake of the Siderophore Triacetylfusarinine C, but Not Fusarinine C, Is Crucial for Virulence of Aspergillus fumigatus
Source: mBio. 2022 Sep 20;13(5):e02192-22. doi: 10.1128/mbio.02192-22 (PMC9600649; doi:10.1128/mbio.02192-22)
Supplement: TABLE S1 [file mbio.02192-22-s0002.docx]

| **Strain** | **Description** | **Reference** |
| --- | --- | --- |
| A1160+ (WT) | CEA10; ∆*kuB^KU80^::pyrG*^+^ | [70] |
| *∆sidA* | A1160+, ∆*sidA*::*six* | this study |
| *∆ftrA* | ATCC46645, ∆*ftrA*::*hph* | [26] |
| *∆ftrA* | *A1160+, ∆ftrA*::*hph* | this study |
| *∆sidA∆ftrA* | ∆*sidA, ∆ftrA*::*hph* | this study |
| *∆mirB* | A1160+, ∆*mirB::hph* | this study |
| *mirB^C^* | ∆*mirB, ∆fcyB::mirB* | this study |
| *∆sidA∆mirB* | ∆*sidA,* ∆*mirB::hph* | this study |
| *∆sidAmirB^C^* | ∆*sidA*∆*mirB, ∆fcyB::mirB* | this study |
| *∆mirD* | A1160+, ∆*mirD::ptrA* | this study |
| *mirD^C^* | ∆*mirD,* ∆*fcyB::mirD* | this study |
| *∆sidA∆mirD* | ∆*sidA, ∆mirD*::*ptrA* | this study |
| *∆sidAmirD^C^* | ∆*sidA∆mirD, ∆fcyB::mirD* | this study |
| *∆sidA∆ftrA∆mirD* | ∆*sidA∆ftrA,* ∆*mirD::ptrA* | this study |
| *∆sidA∆ftrAmirD^C^* | ∆*sidA∆ftrA,* ∆*mirD, ∆fcyB::mirD* | this study |
