# Supplementary material for: Uptake of the Siderophore Triacetylfusarinine C, but Not Fusarinine C, Is Crucial for Virulence of Aspergillus fumigatus
Source: mBio. 2022 Sep 20;13(5):e02192-22. doi: 10.1128/mbio.02192-22 (PMC9600649; doi:10.1128/mbio.02192-22)
Supplement: TABLE S2 [file mbio.02192-22-s0003.docx]

| **Plasmid** | **Description** | **Reference** |
| --- | --- | --- |
| pUC19L | General cloning vector backbone | Thermo Fischer |
| pAN7.1 | Vector with a hygromycin B resistance marker for transforming *Aspergillus* species. | [52] |
| pSK529 | Self-excising hygromycin B resistance marker vector for transforming *Aspergillus* species | [49] |
| pSK275 | *ptrA* resistance gene flanked by *SfiI* sites in pBluescript II KS (syn. pME3024) | [71] |
| p∆*sidA*-rec | pUC19L vector backbone with 3’ and 5’ flanking regions of *A. fumigatus* *sidA* nesting a self-excising hygromycin B resistance cassette | [39] |
| p∆AFU*mirB* | pUC19L vector backbone with 3’ and 5’ flanking regions of *A. fumigatus* *mirB* *rendering* resistance to hygromycin B | this work |
| pMA10 | *A. fumigatus ∆mirD* replacement cassette: ∆*mirD*::P*mirD-hph* flanked by 5’ and 3’ *mirD* homology regions | this work |
| pUC19L-*fcyB* | pUC19L vector backbone with 3’ and 5’ flanking regions of *A. fumigatus* *fcyB* rendering resistance to 5-flucytosine | [54] |
| pMA20 | pUC19L-*fcyB* vector backbone with 3’ and 5’ flanking regions of *A. fumigatus* *mirD* rendering resistance to 5-flucytosine | this work |
| **Primer** | **Sequence** | **Description** |
| MA105 | TTC TGC GTG TCG TTC ATC AGA | *ftrA* 5’ flank |
| MA106 | AAG TTG AGA CTT TGG CCG TA | *ftrA* 3’ flank |
| oAf*mirB*5'_hph.f | TTTAACATTCCCTAGGTACAGAAGTCCAATTG | *hph* 5’ flank |
| oAf*mirB*3'_hph.r | AAGGTCCTACACGCGTTTTATTCTTGTTG | *hph* 3’ flank |
| oAfpUC19L_*mirB*5'.f | AGTGAATTCGAGCTCGGTACACACAATAAAGAGACCTTG | *mirB* 5’ flank |
| oAf*mirB*5'_hph.r | TGTACCTAGGGAATGTTAAATACCGGCTTC | *mirB* 5’ flank |
| oAf*mirB*3'_hph.f | TAAAACGCGTGTAGGACCTTGGATTGCATAC | *mirB* 3’ flank |
| oAfpUC19L_*mirB*3'.r | TTACGCCAAGCTGCATGCCAAGAGAGTGTGAAAAGCTG | *mirB* 3’ flank |
| ohph14 | GAG AGC CTG ACC TAT TGC | *hph* 5’ flank |
| ohph15 | GAGAGCCTGACCTATTGC | *Hph* 3’ flank |
| *fcyB*-1 | CGCTATCCCAGCAATAGAGC | forward 5’ *fcyB* |
| *fcyB*-2RV | TAGTTCTGTTACCGAGCCGGACTGAGTCAATCCCCACCAC | reverse 5’ *fcyB* |
| *fcyB*-3 | GCTCTGAACGATATGCTCCCTGCGGTTTTTGGGTTTTATC | forward 3’ *fcyB* |
| *fcyB*-4RV | CACACTGGGTCTGAAGACGA | reverse 3’ *fcyB* |
| *fcyB*N1 | CAGAGAATTGCCAAGCTGGT | forward nested *fcyB* |
| *fcyB*N2 | GCGGTATGAAACAACGGTCT | reverse nested *fcyB* |
| AfMirBc-FW | CCGGCTCGGTAACAGAACTACTGCCCTCTTGACAACATGA | forward *mirB* 5’-NCR |
| AfMirBc-RV | GGGAGCATATCGTTCAGAGCATCTCATGGGCTGAGGAATG | reverse *mirB 3*’-NCR |
| MA39 | AGCTCGGTACTCGTAATGACCCAGTCATAG | forward *mirD* 5’-NCR |
| MA40 | GTAATCAATTTTCGGGAGAAATAGAACAATG | reverse *mirD* 5’-NCR |
| MA41 | TTCTCCCGAAAATTGATTACGGGATCCC | forward *ptrA*-pSK275 |
| MA42 | AAACCTCACCTCTTGCATCTTTGTTTGTATTATAC | reverse *ptrA*-pSK275 |
| MA43 | AGATGCAAGAGGTGAGGTTTGTCGCTGC | forward *mirD* 3’-NCR |
| MA44 | CTTGCATGCCGTACGAAGAAATCTCGATCAGATAAC | reverse *mirD* 3’-NCR |
