# Supplementary figures and images for: Uptake of the Siderophore Triacetylfusarinine C, but Not Fusarinine C, Is Crucial for Virulence of Aspergillus fumigatus
Source: mBio. 2022 Sep 20;13(5):e02192-22. doi: 10.1128/mbio.02192-22 (PMC9600649; doi:10.1128/mbio.02192-22)

**A**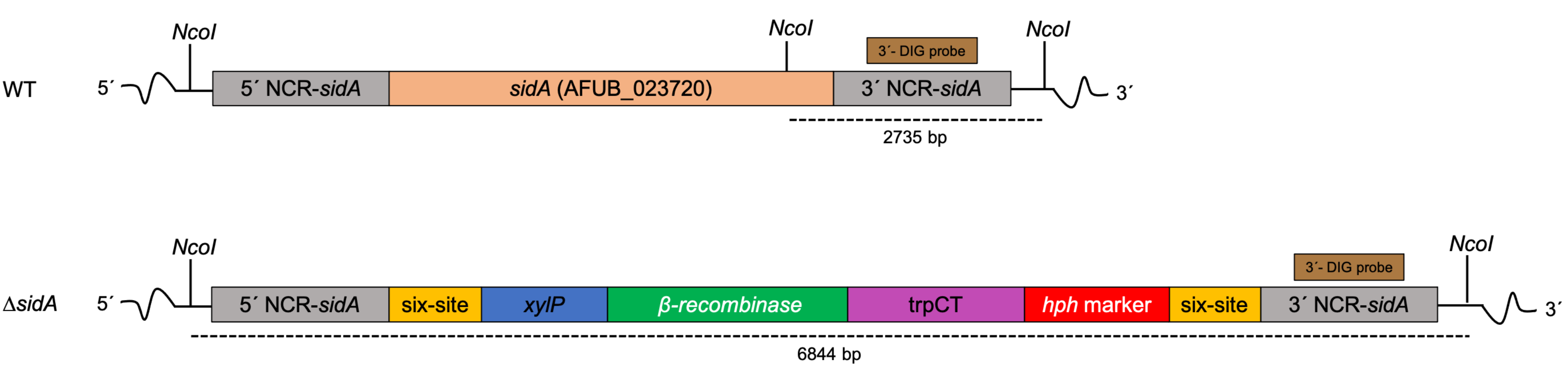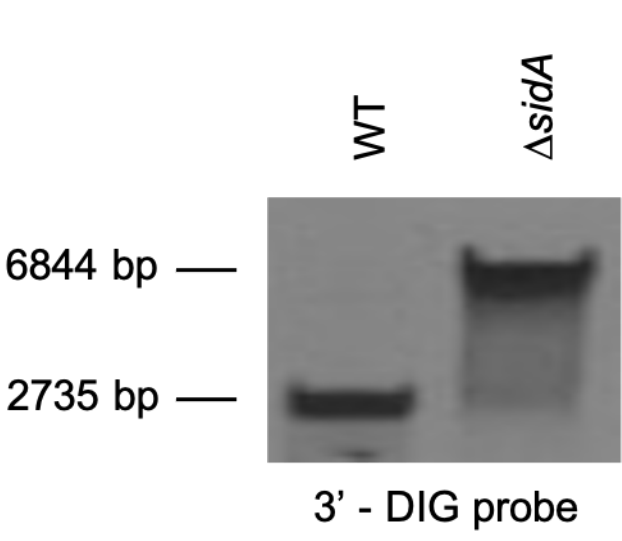**B**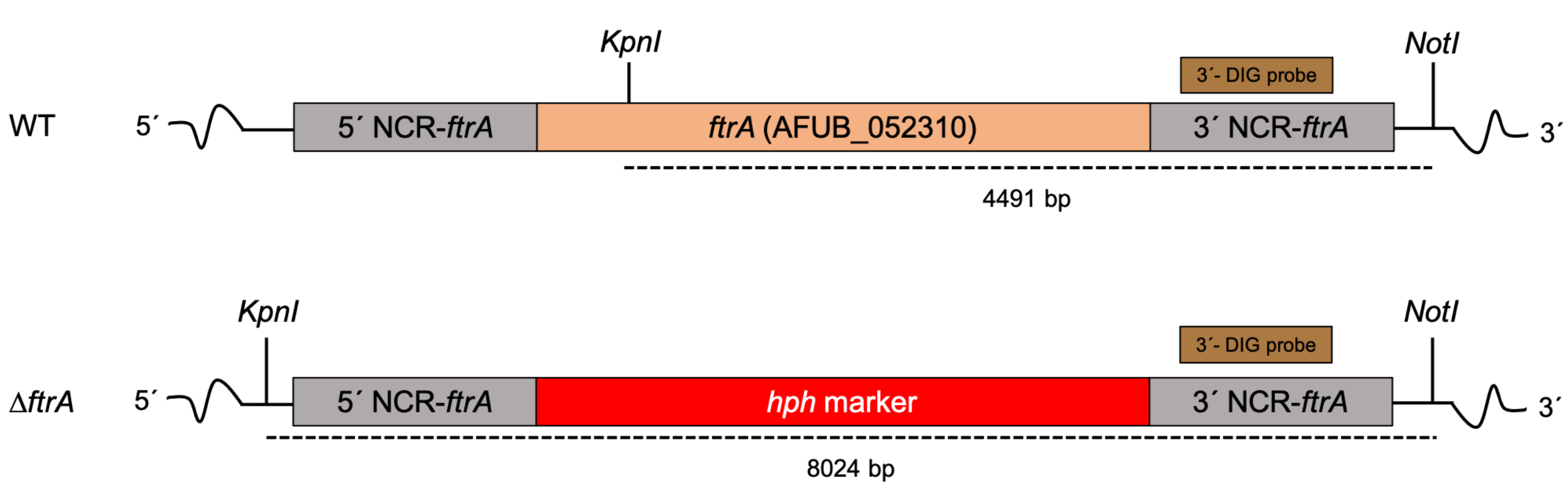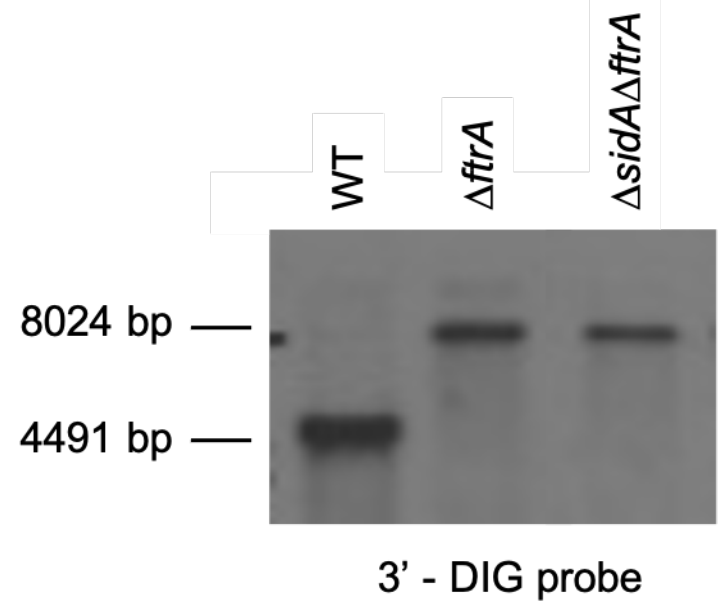**C**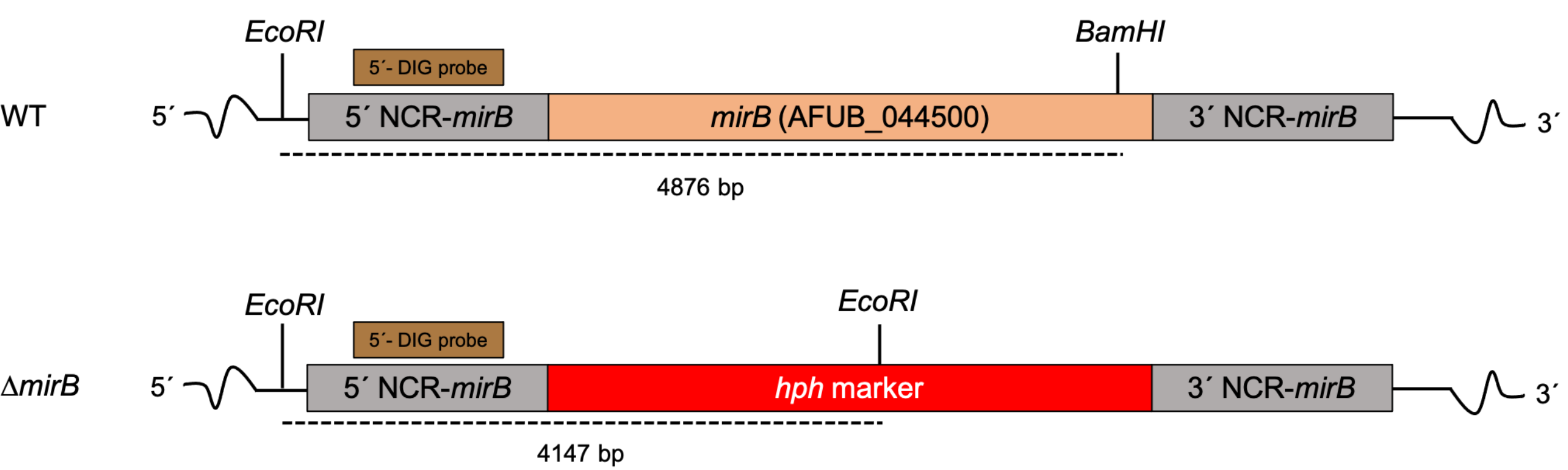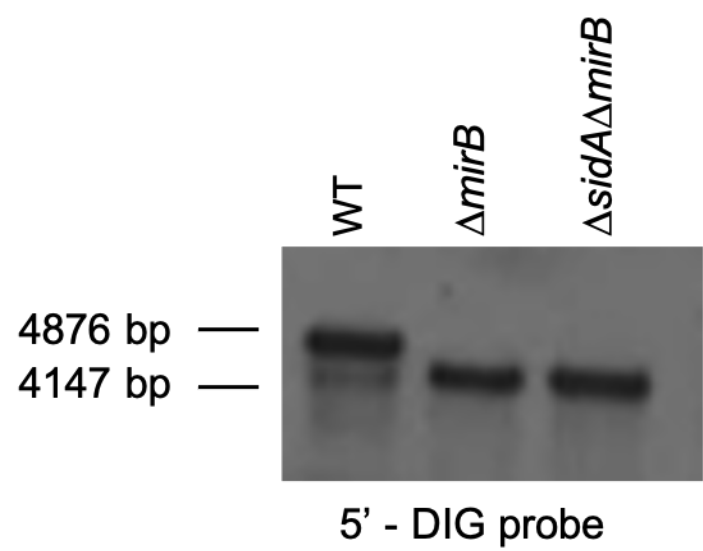**D**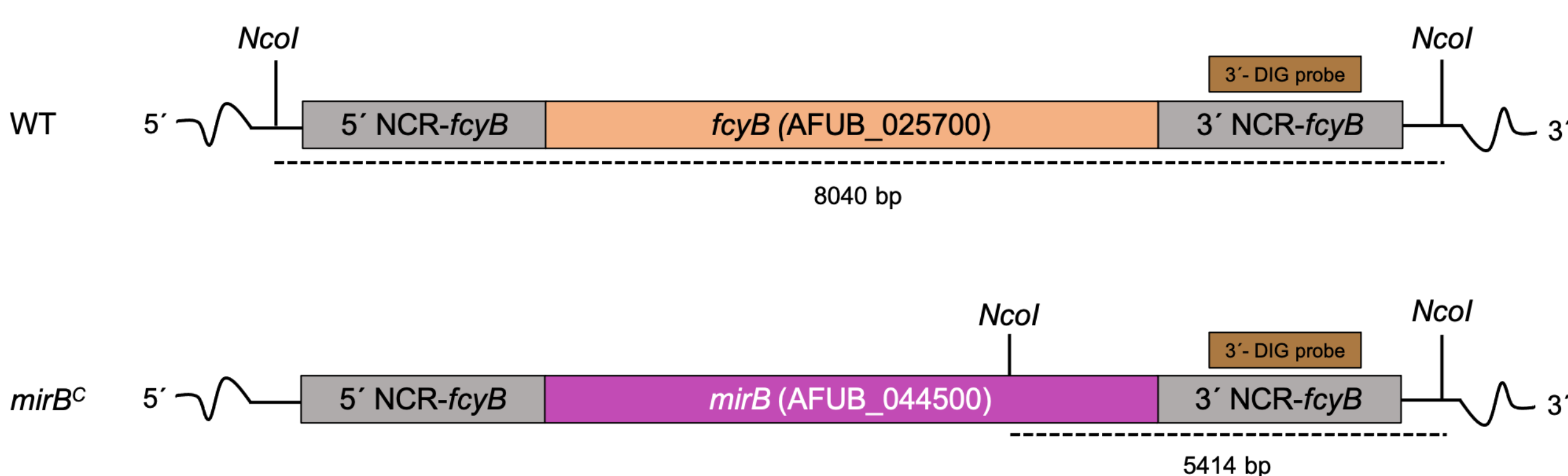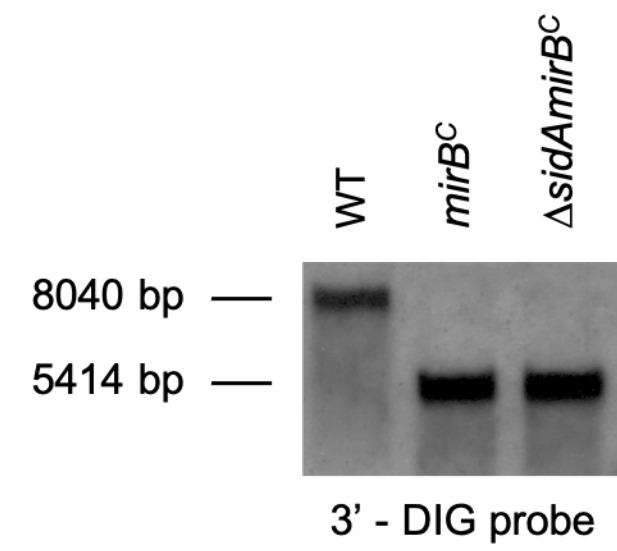**E**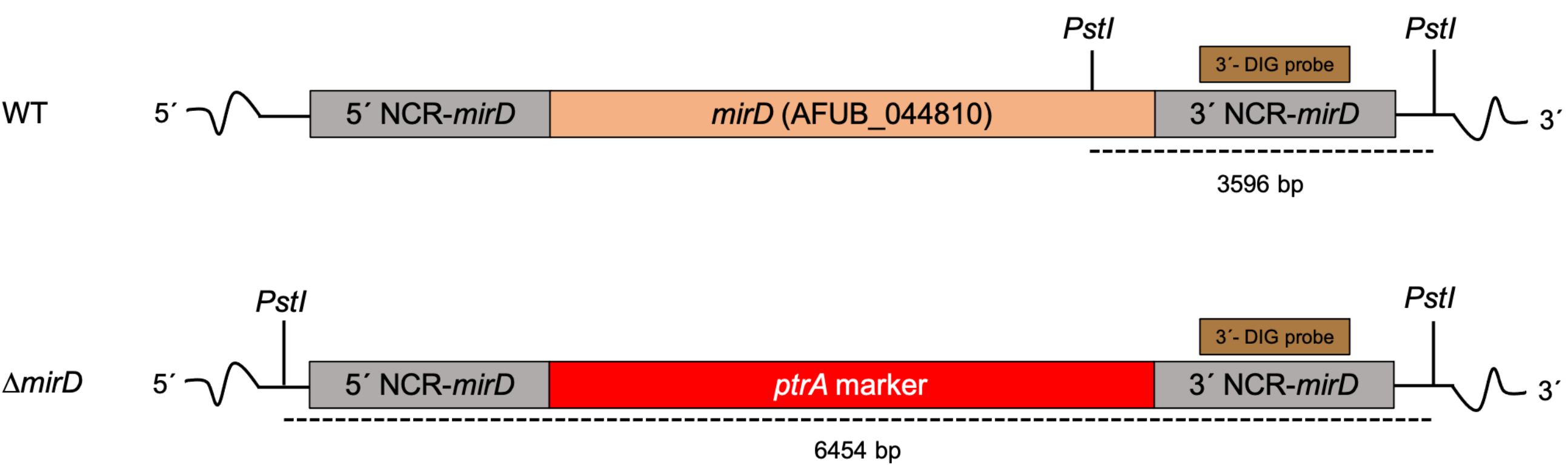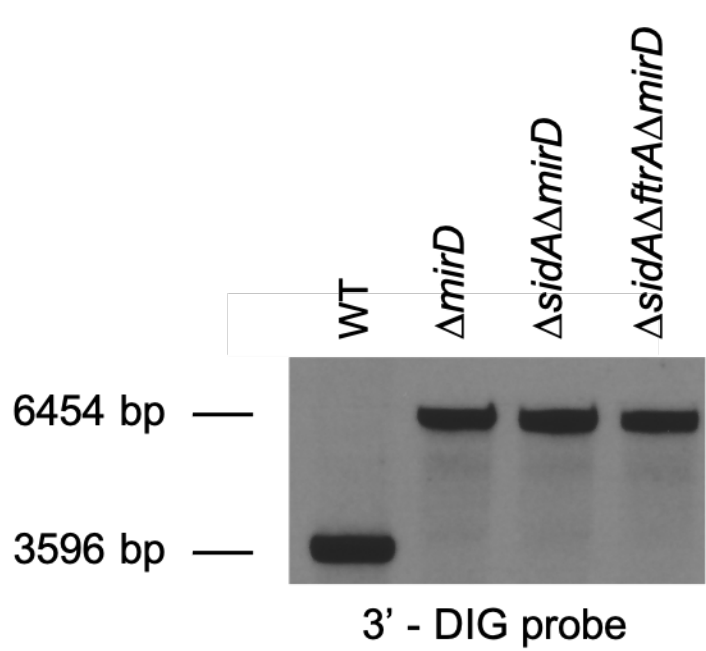**F**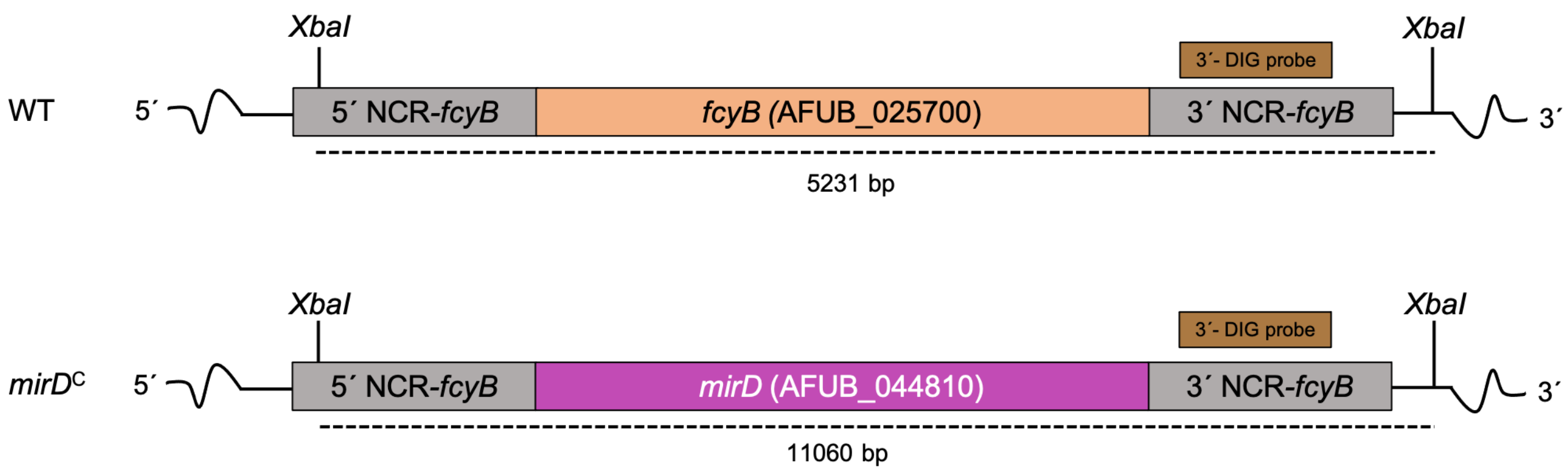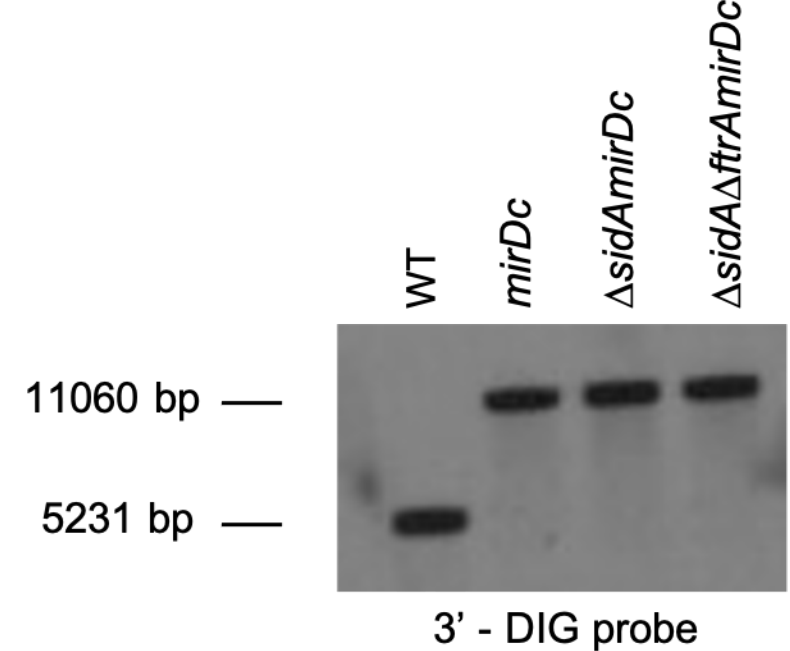

Supplement: FIG S1 [file mbio.02192-22-s0001.pdf]
