# Supplementary material for: Uptake of the Siderophore Triacetylfusarinine C, but Not Fusarinine C, Is Crucial for Virulence of Aspergillus fumigatus
Source: mBio. 2022 Sep 20;13(5):e02192-22. doi: 10.1128/mbio.02192-22 (PMC9600649; doi:10.1128/mbio.02192-22)
Supplement: TABLE S3 [file mbio.02192-22-s0004.docx]

| **Southern blot probe** | **Sequences** | **Gene** |
| --- | --- | --- |
| *3’ sidA* | CTCTGACAACACGATTG  GCACAAAAGAGGACGAG | AFUB_023720 L-ornithine N5-oxygenase |
| *3’ ftrA* | AAACTCTTCACCCAGCCAGC  AGCGTCATTTCTGCGCCA | AFUB_052310 high-affinity iron ion transporter |
| *5’ mirB* | CGC TGA AAA AGG AAG CAG  CTATGGCTGTCCGGTTG | AFUB_044500 siderophore iron transporter |
| *3’ mirD* | TGATGCAGGGGAAATGCTGC  TGATATAGGGAGAGGAGGTAAAGGCG | AFUB_044810 siderophore iron transporter |
| *3’ fcyB* | GCTCTGAACGATATGCTCCCTGCGGTTTTTGGG  CACACTGGGTCTGAAGACGA | AFUB_025700 purine-cytosine permease |
| **Northern blot probe** | **Sequences** | **Gene** |
| *mirB* | AAGCCGAGAAAAAGGGGG  AACCCAGATGAAGCCCAG | AFUB_044500 siderophore iron transporter |
| *mirD* | TGATGCAGGGGAAATGCTGC  TGATATAGGGAGAGGAGGTAAAGGCG | AFUB_044810 siderophore iron transporter |
| *sit2* | GGGTTCTGCTTGTTCTTTGC  CCGCAATGGCAGGGATTCCC | AFUB_090270 siderophore iron transporter |
| *hemA* | ATGATGTCTACGCGGTCC  CAAAGGCAAGACTCCACG | AFUB_053800 *5*-aminolevulinic acid synthase |
